# Taguchi-TOPSIS based optimization of comfort in compression stockings for vascular disorders

**DOI:** 10.1038/s41598-023-43268-7

**Published:** 2023-09-23

**Authors:** Muhammad Nadeem, Hafsa Jamshaid, Rajesh Mishra, Naseer Ahmed, Vijay Chandan, Petr Jirku, Viktor Kolar, Miroslav Muller

**Affiliations:** 1 School of Engineering and Technology, National Textile University, Faisalabad, 37610 Pakistan; 2 Protective Textile Group, National Textile University, Faisalabad, 37610 Pakistan; 3 Department of Material Science and Manufacturing Technology, Faculty of Engineering, Czech University of Life Sciences Prague, 16500 Suchdol, Czech Republic; 4 School of Science, National Textile University, Faisalabad, 37610 Pakistan

**Keywords:** Biomedical engineering, Polymers

## Abstract

Compression stockings/socks are one of the most essential materials to treat vascular disorders in veins. However, the comfort of wearing such stockings over prolonged period of time is a major problem. There is limited research in the area of comfort optimization while retaining the compressional performance. The current work is carried out with an aim to determine the optimum level of the input factors e.g., knitting structure, plaiting yarn linear density and main yarn linear density for achieving desired stretch recovery percentage and thermo-physiological comfort properties of compression socks used in treatment of vascular disorders. Their optimum combination was determined by using Taguchi based techniques for order of preference by similarity to ideal solution i.e., TOPIS. In this study, thickness, areal density, air permeability, thermal resistance, over all moisture management capacity (OMMC), stretch and recovery % were optimized simultaneously by using Taguchi-TOPSIS method. The results showed that linear density of plaiting and main yarn has significant influence on all the comfort related properties for compression stockings/socks. The optimum sample had linear density 20 denier for Lycra covered by 70 denier of nylon 66 in the plaiting yarn. It also suggested 120 denier nylon 66 in the main yarn knitted into a plain single jersey structure. The percentage contribution of the factors i.e., structure, plaiting yarn linear density and main yarn linear density was obtained by using ANOVA which are 7%, 31% and 42% respectively. It is worth mentioning that in case of compression stockings, the main yarn linear density has more significant effect on comfort properties as compared to other independent parameters. The results were verified by experiment, and the accuracy was relatively high (maximum error 8.533%). This study helped to select suitable knit structure with the change of linear densities of plaiting yarn and main yarn for comfortable compression stocking/sock and will fulfill the potential requirement for treatment of venous/vascular disorders. The novel methodology involving TOPSIS method helped in analyzing the cumulative contribution of the input parameters to achieve optimum compression as well as comfort performance. This modern approach is based on contemporary scientific principles and statistical approximations. This study may provide benchmark solutions to complex problems involving multiple interdependent criteria.

## Introduction

Compression stockings/socks are used for physical therapy of venous diseases such as lymphatic insufficiency and varicose veins. They can interfere with the way blood normally flows from the legs up to the heart. These blood flow issues can cause blood to pool/swell in the legs. Compression stockings are specially designed garments used to manage the blood velocity and relieve the venous pressure. Usually these are worn around the ankle, calves and thighs. In the ancient civilization such as Egypt and Rome, many people used such type of garments to treat leg injuries. Compression stockings for health purpose were invented for the first time in 1980s which were capable to reduce the lactate value of blood in the muscles. By using compression garments, a graduated pressure level can be maintained which helps in improving the comfort by avoiding fatigue, swelling and itchiness by the varicose vein related diseases^1–3^. These stockings are recognized as effective nonsurgical treatment that applies pressure to prevent and to treat lower extremities^4^. Compression stockings are known as the first line treatments to manage varicose veins and treat them at initial stage without healing or venous ulceration of legs. It was observed that compression stockings have an effective role in the field of health care and can treat symptoms of deep vein thrombosis (DVT) i.e., initial stage treatment of the varicose diseases^5^. The prevalence of this disease is almost 5% to 45% in adults and is a multi-factorial disease involving positive family history, pregnancy, lifestyle, smoking, obesity and standing long hours at work^6^. It was observed that graduated pressure depends on many factors such as socks size, stiffness, the morphological structure of legs and response of veins^7^. Researchers concluded that compression stocking is required to be smaller in circumference as compared to the leg. Normally the pressure at the ankle position is the highest and it gradually decreases towards the thigh region in order to regulate blood flow^8^. There are different compression standards in countries like Germany, France, UK & USA^9^. The most common standard used is German standard RAL- GZ 387, where class 1 pressure range is 18–21 mm Hg. Researchers also reported that the optimum pressure values which make fastest venous blood flow at different regions such as ankle, calf, knee, lower thigh and upper thigh require pressure levels of 18, 14, 10 and 8 mm Hg respectively^10^.

The parameters such as linear densities of yarns used and the feeding tension have a direct effect on pressure values of medical stockings^11^. Some researchers used different linear densities of polyamide yarn for the evaluation of tensile and elongation properties of stockings^12^. Others observed the effect of different linear densities of inlay yarn, knitting parameters, and relaxation processes on the compression properties^13^. The influence of different parameters including main and inlay yarn linear density on the mechanical properties of compression socks was studied. It was concluded that the inlay and main yarn linear densities impact the extensibility and bursting strength of stockings^14^. The pressure behavior of compression socks developed by 60% polyamide and 40% elastane in plain and rib structure was reported in literature^15^. Researchers observed that main yarn linear density, knitting parameters and fabric structures significantly influence the performance properties of compression stockings^16^. The effect of parameters such as inlay yarn fineness, feeding tension, thickness, stitch density and weight on the pressure behavior of medical stockings was reported^17^. The effect of double covered inlay yarn linear density and insertion density, on the mechanical behavior such as washing and drying cycle shrinkage percentage and compression behavior of the weft knitted orthopedic supports was investigated^18^. The compression of stocking fabric was studied by using polyester and nylon as a main yarn and double covered yarn as inlay yarn. The fabric was developed on the weft knitting machine by using four different linear densities of elastane with three different loop length of main yarn, polyester yarn and nylon yarn. The influence of inlay yarn linear density and ground yarn loop length was observed on the compression, stretch and recovery characteristics by using Kikuhime pressure device and stretch & growth standard respectively. The effect of linear densities of core yarn and inlay yarn on compression and comfort properties was reported^19^. The compression properties were investigated by developing the compression calf sleeves in Pique-knitted structure by using different linear densities of a ground yarn and the draft ratio^20^. The linear density of yarns not only affects the compression properties of fabric but also its comfort properties. Previous studies on yarn linear density have investigated the effect on compression properties, dimensional properties and mechanical properties, but no work has been conducted to investigate the coupled effect of main yarn and plaiting yarn linear density on compression, stretch & recovery as well as thermo-phycological comfort properties.

The stretch and recovery are very important properties for proper fitting of compression socks. Stretching is also directly linked to the comfort of a garment, as it relates to the ease of wearing. The optimum level of stretching is a basic requirement in knitted stockings to support blood flow in the legs and feet. Poor stretching results in a poor fit, leading to performance shortcomings such as discomfort. Compression stockings should be designed in such a way that they maintain a uniform interface pressure gradient over the limb for effective recovery^21^.

The stretch is the amount of extension of the fabric under a determined force, while the elasticity is the fabric’s recovery after stretching, which determines the dimensional stability of an elastic garment.

The stretch percentages can be calculated by using Eq. (1):

1 $$ Fabric\;stretch \, \% \, = \, \left( {B - A} \right)/A \, \times \, 100 $$

where *A* is the original distance between marked points prior to the application of tension, *B* is the distance between benchmark points on the specimen under tension.

For the calculation of the recovery percentages, Eq. (2) can be used:

2 $$ Fabric\;recovery \, \% \, = \, \left( {B - D} \right)/\left( {B - A} \right) \, \times \, 100 $$

where *A* is the original distance between marked points prior to the application of tension, *B* is the distance between benchmarks on the specimen under tension and *D* is the distance between benchmarks after the release of tension.

Compression stockings on the leg generate the pressure with which the stocking presses the leg. Laplace’s law explains how the pressure is exerted by stockings on the limb. It has been reported that pressure exerted by a strip on the surface of a human leg can be determined by Laplace’s law^22^. This law is now widely used to explain and assess the pressure delivered to a limb of a known radius by a fabric under known tension^21,22^. The compression is determined by using Eq. (3):

3 $$ P = T/r $$

where *P* denotes pressure (Pa), *T* is the tension of the compression material on the leg (N m^−1^), *r* is the radius (m) of the limb on which it is applied.

The current research is focused on investigation of thermo-physiological comfort properties of different knit structures such as plain and mock rib structures (longer inlay threads floating at the technical back, forming a mock rib visual effect) e.g., 2 × 2 rib and 3 × 1 rib produced with different linear densities of main and plaiting yarn. It is difficult to choose the appropriate linear density of main and plaiting yarns in order to develop comfortable compression socks that need to be used for a long time during the day especially for venous varicose patients. With the increasing demand of the compression socks for medical purposes, it has become necessary to investigate the required linear density of yarns which should be chosen in order to achieve optimum thermo-physiological comfort characteristics and compression efficiency. Design of experiment (DOE) was used as an effective tool to study the effect of multiple factors on various responses. General DOE is based on L^F^ where L is the level and F is the number of factors. When the number of factors increases, Taguchi method devised by Dr. Genichi Taguchi was used. But when the number of responses increased beyond a certain level, optimization was not possible. To solve this problem, TOPSIS method was used for solving the multi-response Taguchi problems^23,24^. The present study helps to select suitable stockings/socks which are comfortable for the patients by changing the linear densities of plaiting yarn and main yarn with respect to summer conditions. It also fulfills the potential requirement for treatment of venous/vascular disorders by applying adequate graduated compression at different zones of the limb. The novel methodology involving TOPSIS method can help in analyzing the contribution of input parameters to achieve optimum compression as well as comfort performance in medical grade stockings. This modern statistical approach based on scientific principles can provide solutions to several similar problems involving multiple complex criteria.

## Materials and methods

### Materials

**Figure 1:**
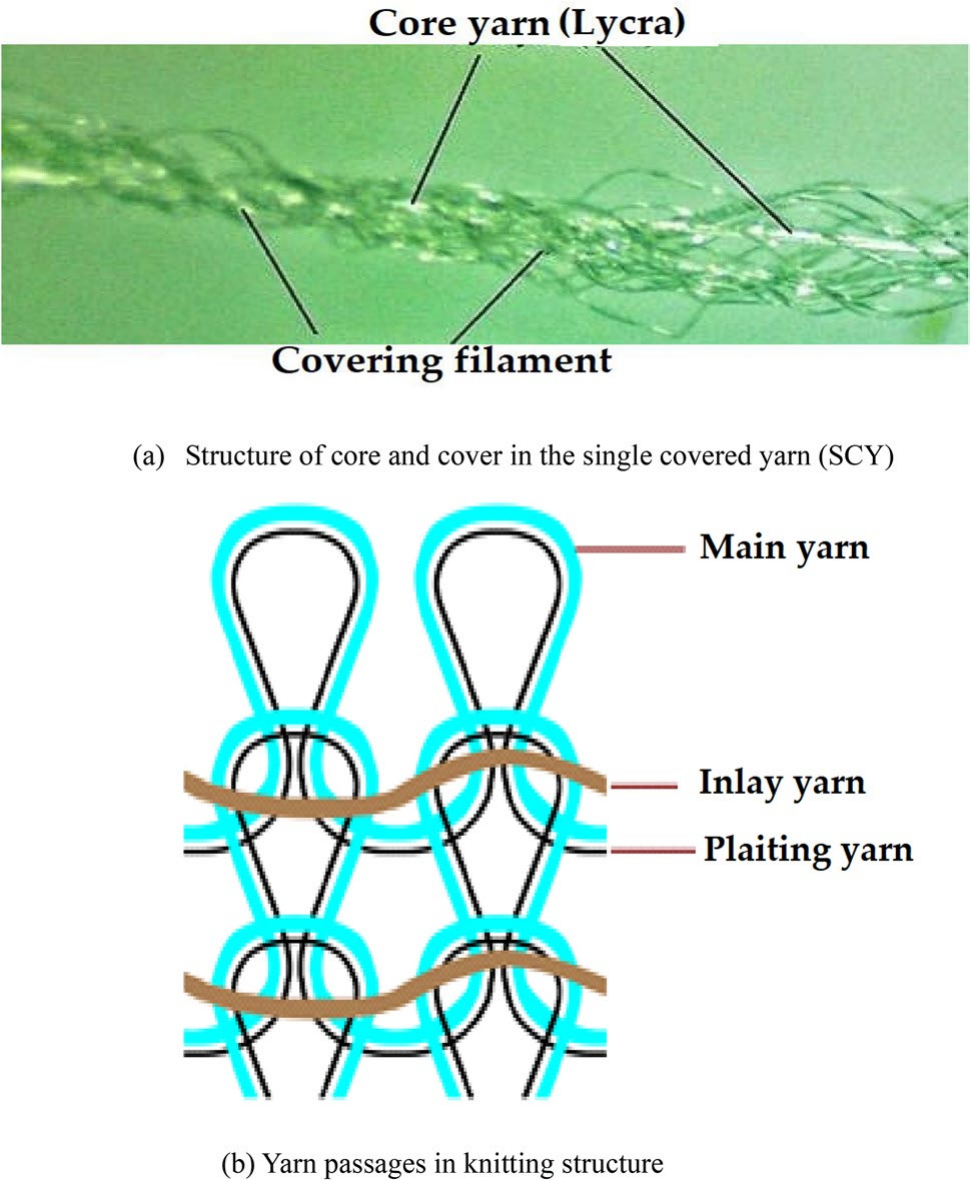
(**a**) Structure of core and cover in the single covered yarn (SCY), (**b**) Yarn passage in knitting structure.

In this stud**y,** for the manufacturing of compression socks, three types of single covered (SCY) plaiting yarns with same type of core yarn (Lycra from DuPont, USA) and linear densities (LD) 20/40, 20/70 and 20/100 denier were used. Three types of main yarn composed of Nylon 6,6 having different linear densities 100, 120 and 140 denier were used. The linear density of inlay yarn, which was a double covered yarn (DC), was maintained at 320 denier for all the samples as shown in Fig. 1. Samples were developed with three different knit structures such as plain, mock rib 2 × 2 and mock rib 3 × 1 by inserting the inlay yarn after one empty course. These structures were selected since they are considered best suited for compression stockings^25^. All the yarns were obtained from Sultani Elasto product Pvt. Ltd., Faisalabad, Pakistan.

### Methods

On the basis of the Taguchi method, there are three factors/optimization objects. These are Structure (A), Plaiting yarn fineness (B) and Main yarn fineness (C). The levels for each of these factors are shown in Table 1.

**Table 1:** Design of experiment (DOE).

Sr. No	Factors	Units	No. of levels	Levels		
1	Structures **(A)**	Nil	3	Plain (P)	Rib 2 × 2 (R1)	Rib 3 × 1 (R2)
2	Plaiting yarn (SCY) **(B)**	Denier/ filaments	3	20/40/12	20/70/24	20/100/36
3	Main yarn (**C)**	Denier/ filaments	3	100/36	120/36	140/48

For example, if the linear density of plaiting yarn is represented as, 20/40/12. The first number 20 represents denier of Lycra core, second number 40 represents denier of covering material and the third number 12 represents the total no. of filaments of cover yarn. In the same way, the main yarn represented by 100/36 means the Nylon 6,6 yarn has 100 denier and 36 is the no. of filaments used in the main yarn.

In total, 9 samples were developed according to Taguchi based TOPSIS method. The sample details are given in Table 2. The fabric structures are shown in Table 3.

**Table 2:** Factors and their levels according to TOPSIS Analysis.

Sample No	Coded Factors			Actual Factors		
	A	B	C	A: Structure	B: SCV-Nylon	C:100%-Nylon
1	1	1	1	Plain	20/40/12	100/36
2	1	2	2	Plain	20/70/24	120/36
3	1	3	3	Plain	20/100/36	140/48
4	2	1	2	Rib 2 × 2	20/40/12	120/36
5	2	2	3	Rib 2 × 2	20/70/24	140/48
6	2	3	1	Rib 2 × 2	20/100/36	100/36
7	3	1	3	Rib 3 × 1	20/40/12	140/48
8	3	2	1	Rib 3 × 1	20/70/24	100/36
9	3	3	2	Rib 3 × 1	20/100/36	120/36

**Table 3:** Structural representations.

Sample code	Sample	Notation	Loop structure	Stocking
P	Plain			
R1	Rib 2 × 2			
R2	Rib 1 × 3			

### Fabric manufacturing

Graduated compression socks were developed on the Merz CC 411 knitting machine (Germany), having four feeders, 12 cm diameter, 24 gauge and 360 needles. The developed samples were marked with points B, B1, and C which are actually the graduated compression points that decrease compression from bottom (ankle) to top (calf) region gradually.

### Processing/washing

All the samples were washed according to Australian guidelines AS-2001:5–2005. A soapy solution was prepared by using mild detergent, the samples were soaked for 5 min and then rubbed softly with each other up to 2–2.5 min. Finally, the samples were rinsed with fresh water. To remove excessive water, samples were pressed one by one. The washed samples were then dried by placing them between layers of absorbing towels for 24 h under standard atmospheric condition (relative humidity (RH) 65 ± 5%, temperature 20 ± 2 °C).

### Physical properties

The linear density of yarns was checked according to ASTM 1059–17, number of filaments were counted by using microscope (Beck London 35,288, Model 47)^26^. Physical properties of the developed sample e.g., Courses per centimeter (CPC), Wales per centimeter (WPC) were checked by using counting glass as per standard method. The areal density (gm^−2^) of the fabric samples was determined by using small grams per square meter (GSM) cutter; model JH-10–36 (Jenhaur Co. Ltd, Taipei, Taiwan) by following the standard ASTM D 3776^27^. Thickness was checked according to the test method ASTM D1777 using thickness tester model 99–0697 (Framincham, MA, USA)^28^. Stretch testing was performed by CETME stretch tester (Reggio Emilia, Italy) by following ASTM D3107-07R19 standard method^29^.

### Compression pressure measurement

Compression pressure can be measured by following two methods which are, in vivo and in vitro. In this research, the pressure on all the developed samples of compression socks was measured by in vitro technique. Testing was performed according to German standard RAL GZ-387 for class 1 in the pressure range 18–21 mm Hg on the Medical Stocking Tester MST MK V, SWL (Swisslastic AG, St. Gallen, Switzerland)^30^. The pressure values are displayed on the screen in different pressure units e.g. mm Hg, mBar and KPa. In this research all values were taken in pressure unit mm Hg. Compression pressure values were checked at the reference points B, B1, and C. The required pressure should be 100% at point B, it should be 70–100% at point B1 and 50–80% at point C for an appropriate blood velocity to treat the varicose disease^24^.

### Thermo-physiological comfort characteristics

Air permeability was tested by following ISO-9237 standard on the Air permeability tester (M021A, SDL Atlas) by setting the pressure at 100 Pa^31^. The thermal resistance of the samples was checked by ISO-11092 standard on Alambeta instrument (Czech Republic)^32^. The overall moisture management capacity (OMMC) of all samples was tested as per standard AATCC-195 by using Moisture management tester (M290, SDL Atlas)^33^. For each property, ten measurements were taken per sample, and their average was calculated. All the experiments were performed at standard atmospheric condition i.e., 65 ± 2% relative humidity and 20 ± 2 °C temperature according to standard ISO-139^34^.

### Data analysis technique

Technique of order of preference by similarity to ideal solution (TOPSIS) was developed in 1980 for multi criteria decision matrix (MCDM). TOPSIS method is based on the Euclidean distance measured from positive ideal solution (PIS) and negative ideal solution (NIS) for alternatives. This technique provides optimum solution as the closest distance to PIS and farthest distance to NIS^23–25^. The PIS is the solution that shows maximization of the benefits while the NIS maximizes the cost criteria and minimizes benefits. The Taguchi method output is transformed into Signal to Noise (S/N) ratio. The signal values show the real output that has to be measured while the noise values represent the variance. For better results, signal rate should be greater than the noise rate. The lowest, highest, and nominal values are considered. In this experimental study, the stockings were considered for summer season. Therefore, the highest OMMC, highest air permeability, highest stretch and recovery percentage was considered. The lowest thermal resistance, thickness and areal density are suitable for optimum comfort of compression socks during summer. The order of preference for this analytical technique was selected as (1) OMMC, (2) air permeability, (3) stretch percentage, (4) recovery percentage, (5) thermal resistance, (6) thickness and (7) areal density respectively.

## Results and discussion

### Compression pressure

**Figure 2:**
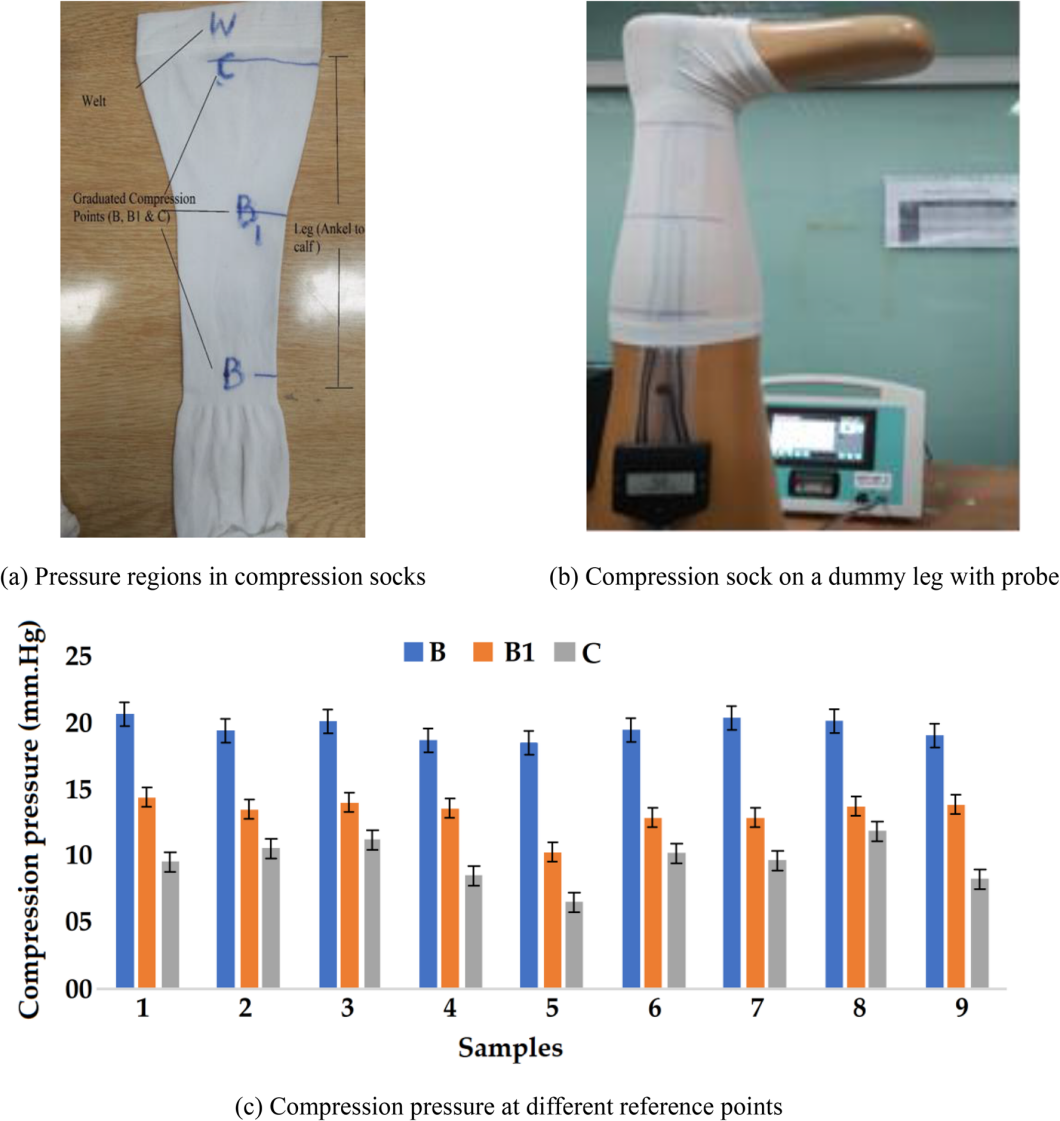
Pressure measurement, (**a**) Pressure regions in compression socks**,** (**b**) Compression sock on a dummy leg with probe and (**c**) Compression pressure at different reference points.

Compression pressure measurement of all the samples in class 1 according to German standard RAL GZ 387 is shown in Fig. 2a,b. The pressure values at different reference points (B, B1 & C) are compared in Fig. 2c. It can be seen that pressure from point B to point C decreases gradually to regulate the blood flow.

**Figure 3:**
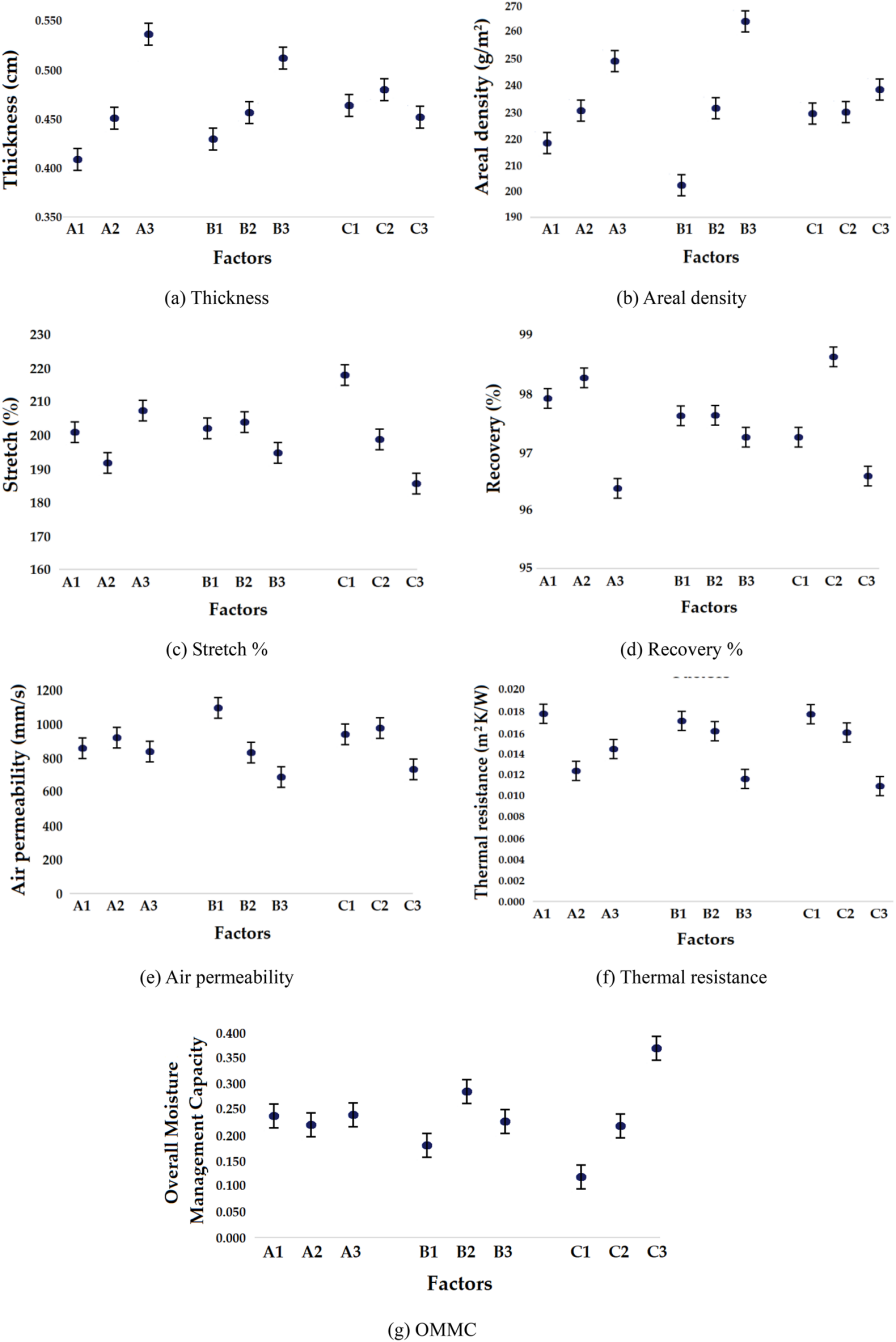
Physical and comfort related properties of developed compression stockings/socks.

The trends are uniform regarding the decrease of compression pressure from point B to B1 to C. For all the 9 samples developed, measurements of comfort related attributes e.g., thickness, areal density, stretch and recovery %, air permeability, thermal resistance and OMMC were carried out. The average value for each characteristic along with the standard deviation (± SD) is given in Table 4. Further analysis of comfort properties among the samples based on the levels of factors mentioned in Table 1 are given in Fig. 3a–g.

**Table 4:** Samples and their responses detail.

Samples	Responses						
Sr. No	Y1: Thickness (cm)	Y2: Areal density (g/m^2^)	Y3: Air permeability (AP) (mm/sec)	Y4: Thermal resistance (TR) (m^2^ K/W)	Y5: OMMC	Y6: Stretch (%)	Y7: Recovery (%)
1	0.386 ± 0.045	186.17 ± 7.45	1044.00 ± 41.20	0.027 ± 0.001	0.090 ± 0.01	223.07 ± 6.55	96.55 ± 4.15
2	0.410 ± 0.023	214.60 ± 11.14	1001.67 ± 46.24	0.016 ± 0.001	0.267 ± 0.01	207.15 ± 5.47	100.00 ± 5.55
3	0.430 ± 0.047	251.25 ± 12.41	564.00 ± 19.14	0.009 ± 0.001	0.360 ± 0.01	176.31 ± 4.45	97.01 ± 4.12
4	0.415 ± 0.036	190.70 ± 8.42	1249.00 ± 29.42	0.015 ± 0.001	0.141 ± 0.01	186.63 ± 5.12	99.99 ± 5.05
5	0.441 ± 0.041	237.07 ± 10.21	608.33 ± 20.36	0.014 ± 0.001	0.450 ± 0.01	180.10 ± 6.05	96.29 ± 3.25
6	0.491 ± 0.035	262.25 ± 12.42	881.67 ± 24.34	0.008 ± 0.001	0.081 ± 0.01	208.82 ± 7.01	98.59 ± 5.40
7	0.485 ± 0.042	228.07 ± 14.41	1005.00 ± 41.53	0.008 ± 0.001	0.303 ± 0.01	200.02 ± 6.41	96.43 ± 4.85
8	0.510 ± 0.047	240.00 ± 10.46	875.33 ± 32.11	0.018 ± 0.001	0.160 ± 0.01	223.07 ± 7.74	96.55 ± 5.40
9	0.611 ± 0.044	279.49 ± 15.72	601.00 ± 20.54	0.017 ± 0.001	0.253 ± 0.01	200.02 ± 5.45	96.15 ± 4.63

The variation in linear density of yarns greatly influences all physical and thermo-physiological comfort properties. The thickness results show an increasing trend from plain to rib 2 × 2 and rib 3 × 1 structure as the linear density increases. It is observed from the Table 4 and Fig. 3a that the thickness of plain structures is relatively lower as compared to rib 2 × 2 and rib 3 × 1. This is because of higher compactness in plain knitted fabric as a result of the loop structure. With higher linear density of plaiting yarn, the thickness increases in all the knitted structures (plain, rib 2 × 2 and rib 3 × 1). With increasing liner density of main yarn, the thickness increases from finer to coarser yarn. In the similar manner, the areal density (GSM) also increases from plain to rib structures as shown in Table 4 and Fig. 3b. This increase in GSM among the structures is due to accumulation of yarn at the tucking zone of rib knitted structures. The inlay yarn feeding consumes more yarn and causes increase of GSM in rib 2 × 2 and rib 3 × 1^22^. With the increase of overall linear density from finer to coarser yarn of the plaiting yarn, the GSM increases.

### Stretch and recovery %

From the results in Fig. 3c, it can be seen that there is significant influence of linear density of plaiting yarn and main yarn on stretch and recovery percentage. Finer linear density leads to higher stretch % as compared to coarser linear density because finer yarn ensures loose structure as compared to coarser yarn^29^. From the results, it was observed that the stretch % decreases from 223.07% to 176.31% for sample 1 and 3 respectively, which indicate the highest and lowest values. This decreasing trend is observed within the same structure and among the different structures. As tuck stiches are involved in 2 × 2 and 3 × 1 rib knits, stretch % decreases. A tucked wale is less stretchable than the floating wale. In the rib knit structures, presence of tuck stitches also increases recovery %. The tucked loop enables a more stable structure with enhanced shape retention in rib structures. It was observed that due to accumulation of yarn in tuck stitches, the shrinkage % increases which improves recovery %. Recovery results in Fig. 3d show that as the plaiting yarn becomes coarser, especially from 20/40/12 to 20/70/24 and then to 20/100/36, it leads to lower recovery %. It may be due to cumulative effect of higher number of filaments in the yarn assembly and elastane core, which generates more inter filament friction to resist extension and thus improves recovery. Recovery% was observed to increase from 96.18% to 100% as per results which is suitable for compression socks/stockings in order to retain their properties even after washing^30^. The variation of physical and comfort related properties are shown in Fig. 3a–g.

### Thermo-physiological comfort properties

The effect of knit structure on air permeability is not very significant as visible in Fig. 3e. However, the air permeability decreases as the linear density of plaiting yarn increases in all the structures. Finer the yarn, less is the resistance to the air flow due to more open spaces among fibers. This significant impact of plaiting yarn is due to Lycra which consolidates the covering. The cover yarn having finer linear density wrapped over the core filament in most parallel form facilitates easier passage of air. As covering fiber/filament content increases in coarser yarns e.g., from 12 to 24, it causes hindrance in the passage of air due to non-parallelization of fibers and inter-fiber entanglement which reduces the air permeability. It was investigated by several researchers that by using coarser linear density of yarn for knitted fabrics, the air permeability decreases significantly^26^. It slightly increases as the main yarn linear density is higher however, decreases again due to more compactness of the filaments^32^. The fineness of the used yarns has significant influence on the thermal resistance. By increasing the liner density of main yarn and plaiting yarn, thermal resistance increases within the same structure and among the different structures as visible in Fig. 3f. The coarser yarns facilitate a fabric with higher porosity and higher thickness. These factors are responsible for a higher thermal resistance^33^. OMMC is considered as the ability of fabric to remove the moisture away from the skin and transport it for evaporation. As the number of miss stiches increases (in rib knit), the OMMC also improves as can be seen from the Fig. 3g. From the graphical analysis it was observed that as main yarn becomes coarser, OMMC value increases. This may be due to a greater number of filaments that provide maximum capillary effect for moisture transmission that leads to higher OMMC value. As plaiting yarn becomes coarser, OMMS first increases and then decreases. This decrease may be due to difference of number of filaments which impacts the capillary action.

### Taguchi based TOPSIS analysis

The Taguchi based TOPSIS analysis involved 9 runs with 7 responses. By giving the highest, lowest and nominal preferences to the responses (Characteristics) as per analysis technique which prefers closest value to ideal solution (Ci), positive and negative ideal solution values decide rank of the sample. For selection of best quality characteristics, a comparison was done among all samples by normalizing signal to noise (S/N) ratio. Quality characteristics define some responses need lower the better and some need higher the better or nominal value the better conditions. In this experimental study, the quality factor OMMC should be higher the better, air permeability (mm/s) higher the better, stretch and recovery percentage higher the better, the thermal resistance (m^2^K/W) lower the better, GSM and thickness (cm) are considered lower the better for the summer application of compression stockings. The signal to noise ratios and normalized signal to noise ratios are given in Tables 5 and 6.

**Table 5:** Signal to Noise (S/N) Ratio.

Samples	S/N ratio						
Sr. No	Y1: Thickness (cm)	Y2: Areal density (g/m^2^)	Y3: Air permeability (AP) (mm/sec)	Y4: Thermal resistance (TR) (m^2^ K/W)	Y5: OMMC	Y6: Stretch %	Y7: Recovery %
1	0.652	− 45.398	60.373	31.369	− 20.916	46.969	39.695
2	0.574	− 46.633	60.014	35.906	− 11.485	46.326	40.000
3	0.541	− 48.002	55.025	40.537	− 8.874	44.926	39.736
4	0.456	− 45.607	61.931	36.465	− 17.016	45.420	39.999
5	0.512	− 47.497	55.681	37.036	− 6.940	45.110	39.672
6	0.146	− 48.374	58.906	42.270	− 21.867	46.396	39.877
7	0.264	− 47.161	60.043	41.902	− 10.365	46.021	39.684
8	− 0.172	− 47.604	58.843	34.927	− 15.952	46.969	39.695
9	− 0.452	− 48.927	55.577	35.374	− 11.931	46.021	39.659

**Table 6:** Normalized signal to noise (S/N) ratio.

Samples	Normalized S/N ratio						
Sr. No	Y1: Thickness (cm)	Y2: Areal density (g/m^2^)	Y3: Air Permeability (AP) (mm/sec)	Y4: Thermal Resistance (TR) (m^2^ K/W)	Y5: OMMC	Y6: Stretch (%)	Y7: Recovery (%)
1	0	0	0.774	1.000	0.064	1.000	0.106
2	0.128	0.350	0.722	0.584	0.696	0.685	1.000
3	0.232	0.738	0.000	0.159	0.870	0.000	0.227
4	0.154	0.059	1.000	0.532	0.325	0.242	0.998
5	0.290	0.595	0.095	0.480	1.000	0.090	0.037
6	0.524	0.843	0.562	0.000	0.000	0.719	0.638
7	0.494	0.500	0.727	0.034	0.771	0.536	0.073
8	0.604	0.625	0.553	0.674	0.396	1.000	0.107
9	1	1	0.080	0.633	0.666	0.536	0
Weightage %	4%	6%	20%	8%	32%	16%	14%

Weightage percentages are mentioned in the table of normalized signal to noise ratio. Further the weighted and normalized decision matrix is given in Table 7.

**Table 7:** Weighted and normalized decision matrix.

Samples	Weighted and Normalized Decision Matrix						
Sr. No	Y1: Thickness (cm)	Y2: Areal density (g/m^2^)	Y3: Air permeability (AP) (mm/sec)	Y4: Thermal resistance (TR) (m^2^ K/W)	Y5: OMMC	Y6: Stretch (%)	Y7: Recovery (%)
1	0.0000	0.0000	0.1586	0.0827	0.0203	0.1640	0.0144
2	0.0045	0.0200	0.1479	0.0483	0.2223	0.1124	0.1363
3	0.0082	0.0423	0.0000	0.0131	0.2782	0.0000	0.0309
4	0.0054	0.0034	0.2048	0.0440	0.1039	0.0397	0.1361
5	0.0102	0.0341	0.0195	0.0397	0.3196	0.0148	0.0051
6	0.0185	0.0483	0.1151	0.0000	0.0000	0.1180	0.0870
7	0.0174	0.0286	0.1488	0.0028	0.2463	0.0880	0.0100
8	0.0213	0.0358	0.1132	0.0557	0.1266	0.1640	0.0145
9	0.0353	0.0573	0.0164	0.0523	0.2127	0.0880	0.0000

The positive ideal solution (Si +) and negative ideal solution (Si–), closeness to ideal solution (Ci) and the rank are given in Table 8. Ranking of the best sample with respect to summer application is also given in Table 8.

**Table 8:** Si + , Si– and Ci.

Sr. No	Si +	Si–	Ci	Rank
1	0.3367	0.2392	0.4153	8
2	0.1346	0.3256	0.7075	1
3	0.2893	0.2901	0.5007	5
4	0.2530	0.2794	0.5249	4
5	0.2769	0.3252	0.5401	3
6	0.3426	0.2048	0.3741	9
7	0.1772	0.3133	0.6388	2
8	0.2555	0.2395	0.4839	6
9	0.2802	0.2328	0.4537	7

Table 9 shows the ANOVA results.

**Table 9:** ANOVA results.

Factor	Sum of squares	Degrees of freedom	Mean sum of squares	F-Test	Contribution (%)
A	0.0061	2	0.0031	0.3637	7
B	0.0276	2	0.0138	1.6406	31
C	0.0373	2	0.0186	2.2168	42
Error	0.0168	2	0.0084		19
Total	0.0878	8			

**Figure 4:**
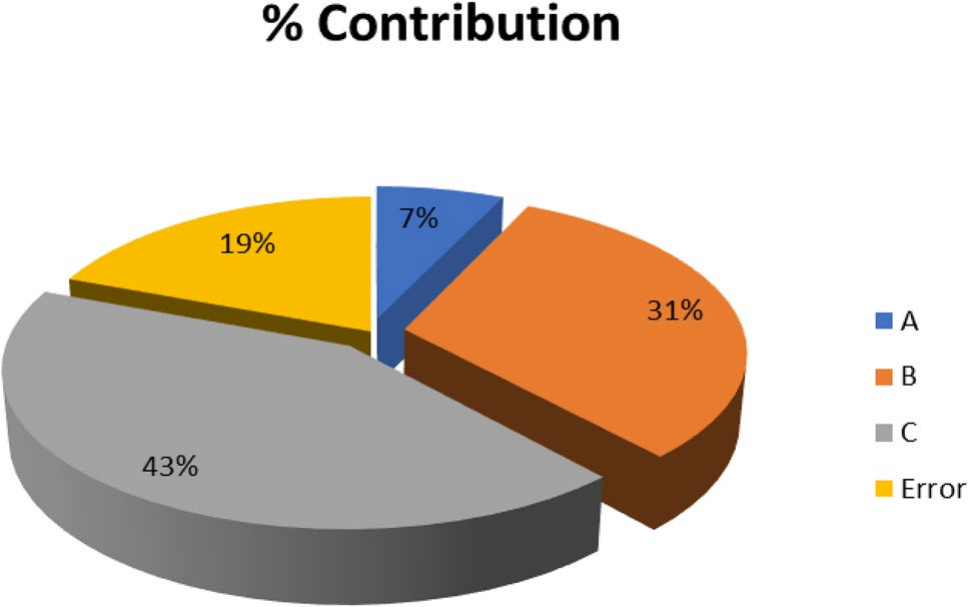
Percentage contribution of different factors.

The contribution of individual factors is shown in Fig. 4.

The confirmatory experimental results are given in Table 10.

**Table 10:** Confirmatory experimental results.

Responses	A1B2C2	Confirmatory test	Error	|Error|	Error (%)
Y1: Thickness (cm) (L)	0.82	0.854	− 0.034	0.034	4.1463
Y2: GSM (g m^−2^) (L)	214.6	216.3	− 1.7	1.7	0.7922
Y3: AP (mm/sec) (H)	1001.666667	1000	1.66666667	1.666667	0.1664
Y4: TR (m^2^ K/W) (L)	0.016	0.0159	1E-04	1E-04	0.6250
Y5: OMMC (H)	0.266666667	0.27	− 0.0033333	0.003333	1.2500
Y6: Stretch % (H)	207.1466667	206	1.14666667	1.146667	0.5536
Y7: Recover % (H)	100	101	− 1	1	1.0000
Total Error %					8.5335

*(H) indicates, higher the better., (L) indicates, lower the better.

The main effects in TOPSIS are shown in Table 11.

**Table 11:** Main effects in TOPSIS.

Factors	1	2	3	Max
A	0.54116572	0.479702	0.52545847	0.541166
B	0.52631877	0.577168	0.44283971	0.577168
C	0.42441777	0.562045	0.55986334	0.562045

(A = Structures, B = Single Covered Nylon or plaiting yarn, C = Main yarn).

From the results it was observed that the sample 2, which is a plain knitted structure having plaiting yarn 20/70/24 (Lycra denier 20/nylon denier 70/no. of filaments 24) and main yarn 120/36 (Nylon 120 denier/no. of filaments 36) is the best sample and ranked at 1^st^. Sample 7 at 2^nd^ rank and sample 5 at the 3^rd^ rank are considered the best among all the 9 samples. This sample preference is for the compression stockings applicable for summer season or higher temperature and humidity conditions.

## Conclusions

This research was intended to develop comfortable compression stockings/socks by using various linear densities of main yarn and plaiting yarn with constant inlay yarn linear density for the graduated compression socks class 1. The compression pressure of all the samples falls in the range of class 1 i.e., within 18–22 mm Hg which is necessary to regulate the blood flow to treat initial venous disease. It is very challenging for the manufacturer to select appropriate linear density of main yarn, plaiting yarn and the knit structure that meets all functional needs of the end user (patient). In this work, multi response optimization method such as Taguchi based TOPSIS analysis technique was applied. It was observed that the sample 2 performed as the best (1^st^ rank) followed by sample 7 at 2^nd^ place and sample 5 was ranked as the 3^rd^. This optimization method suggests the samples having plain structure developed by 20 denier Lycra yarn with covering of 70 denier nylon as a plaiting yarn and 120 denier nylon as a main yarn, is the best sample that fulfills the criteria of comfortable compression socks. It is expected that this research work will support in development of comfortable compression socks with low cost and high production rate that will prove beneficial for the growth of medical textiles in treatment of vascular disorders/venous diseases. This study helped to select suitable knit structure with the change of linear densities of plaiting yarn and main yarn for comfortable compression stocking/sock and will fulfill the potential requirement for treatment of venous/vascular disorders. The novel methodology involving TOPSIS method helped in analyzing the cumulative contribution of the input parameters to achieve optimum compression as well as comfort performance. This modern approach is based on contemporary scientific principles and statistical approximations. This study can provide best solutions to complex problems involving multiple interdependent criteria.

## Data Availability

The data used to support the findings of this study is included within the article.
